# Measures of Association for Identifying MicroRNA-mRNA Pairs of Biological Interest

**DOI:** 10.1371/journal.pone.0029612

**Published:** 2012-01-11

**Authors:** Vivek Jayaswal, Mark Lutherborrow, Yee Hwa Yang

**Affiliations:** 1 School of Mathematics and Statistics, University of Sydney, Sydney, New South Wales, Australia; 2 Centre for Mathematical Biology, University of Sydney, Sydney, New South Wales, Australia; 3 Department of Haematology, St. Vincent Centre for Applied Medical Research, Darlinghurst, New South Wales, Australia; University of North Carolina at Charlotte, United States of America

## Abstract

MicroRNAs are a class of small non-protein coding RNAs that play an important role in the regulation of gene expression. Most studies on the identification of microRNA-mRNA pairs utilize the correlation coefficient as a measure of association. The use of correlation coefficient is appropriate if the expression data are available for several conditions and, for a given condition, both microRNA and mRNA expression profiles are obtained from the same set of individuals. However, there are many instances where one of the requirements is not satisfied. Therefore, there is a need for new measures of association to identify the microRNA-mRNA pairs of interest and we present two such measures. The first measure requires expression data for multiple conditions but, for a given condition, the microRNA and mRNA expression may be obtained from different individuals. The new measure, unlike the correlation coefficient, is suitable for analyzing large data sets which are obtained by combining several independent studies on microRNAs and mRNAs. Our second measure is able to handle expression data that correspond to just two conditions but, for a given condition, the microRNA and mRNA expression must be obtained from the same set of individuals. This measure, unlike the correlation coefficient, is appropriate for analyzing data sets with a small number of conditions. We apply our new measures of association to multiple myeloma data sets, which cannot be analyzed using the correlation coefficient, and identify several microRNA-mRNA pairs involved in apoptosis and cell proliferation.

## Introduction

 MicroRNAs (miRNAs) are small (∼22 nt) non-protein coding RNAs that are involved in the post-transcriptional regulation of mRNA expression. The miRNAs are of immense biological significance, e.g. changes in miRNA expression have been linked to cancer [1]–[4], and, over the past two decades, numerous studies have focused on miRNAs. The studies on miRNAs can be broadly grouped into two categories – identification of miRNAs as molecular markers for better prognosis/diagnosis [5], [6] and understanding the role of miRNAs in transcription regulation [7]–[11]. In this paper, we focus on the latter category and introduce new methods for obtaining insights into a miRNA's regulatory role.

 The identification and validation of a regulatory miRNA requires a knowledge of its target mRNAs and, initially, computational algorithms such as TargetScanS [12], PicTar [13] and miRanda [14] were used to obtain the putative miRNA-mRNA pairs based on sequence data. Although, for every miRNA, these algorithms suggested a potential pairing with several hundred mRNAs, the number of genuine pairs was much lower (≈50%) [15]. Even if a pair is genuine, it may not be of interest in a particular biological condition because the regulatory miRNAs vary from one condition to another. For example, the miRNAs that are regulatory in lung cancer may not be regulatory in pancreatic cancer. Therefore, the computational algorithms are not sufficient to obtain the pairs of interest under different biological conditions.

Over the past decade, several methods [16]–[19] have been developed that combine the results of computational algorithms with mRNA expression data. While these methods are suitable for identifying the potentially regulatory miRNAs, they cannot be used to obtain the miRNA-mRNA pairs for experimental validation. This is because a miRNA-mRNA pair is of potential biological interest only if there is an association between the expression levels of the relevant miRNA and mRNA. Consequently, integrative methods that combine the results of target-prediction algorithms with both mRNA and miRNA expression data have become popular. While some of these integrative methods focus on the identification of miRNA-mRNA pairs [20]–[25], others focus on the identification of miRNA-mRNA modules, i.e. groups of miRNAs that co-regulate groups of mRNAs [26]–[30]. Broadly speaking, the integrative methods employ a three step procedure as described below:

1. Identification of differentially expressed (DE) miRNAs and mRNAs: An expression data set corresponds to multiple conditions and one of these, e.g. healthy state, is selected as the reference. Next, the miRNAs and mRNAs that are DE, with respect to reference, in at least one of the conditions are identified.
2. Selection of putative miRNA-mRNA pairs: A miRNA-target prediction algorithm is used to obtain the DE mRNAs that are putative targets of one or more of the DE miRNAs.
3. Identification of statistically significant miRNA-mRNA pairs: The miRNA and mRNA expression profiles are used to obtain the statistical significance of the pairs identified in Step 2.The statistically significant pairs are considered to be of potential biological interest and are usually selected for further experimental studies or validation. Unless specified otherwise, in the rest of this paper, significance implies statistical significance.

The identification of statistically significant pairs (Step 3) is a challenging task and has two components – the selection of an appropriate association measure and the determination of its significance. Most studies on the identification of miRNA-mRNA pairs of interest utilize the correlation coefficient [20], [22]–[25], [27] as a measure of association and several methods have been proposed to obtain its significance. Lionetti et al. [20] used the correlation coefficient to rank the miRNA-mRNA pairs such that the pair with the strongest negative correlation was ranked one. Next, the authors chose an arbitrary cut-off of 3% and considered the top 3% of the pairs (in terms of rank) to be of potential biological significance. A more formal approach for identifying the miRNA-mRNA pairs of biological interest is based on a test of significance. If the distribution of correlation coefficients is known, then this distribution can be used to perform the significance test, e.g. Gutierrez et al. [24] performed a test of significance under the assumption that the distribution was bivariate normal. If the distribution is unknown, then a permutation test [25], [27] is more appropriate for obtaining the significance of association.

The use of correlation coefficient as an association measure is appropriate only if every individual in the study is used to obtain both miRNA and mRNA expression profiles. In other words, the use of correlation coefficient requires the miRNA and mRNA expression data to be matched. Moreover, the matched data have to be available for several biological conditions. For a small number of conditions, the correlation coefficient-based measure can be very noisy and for the extreme case where the number of conditions is just two, this measure is not meaningful. Therefore, there is a need for new measures of association to identify the miRNA-mRNA pairs of interest using a small number of biological conditions. To this end, we introduce an association measure that enables the identification of significant miRNA-mRNA pairs using matched expression data for just two conditions.

The number of biological conditions can be increased by combining multiple miRNA and mRNA studies. In theory, the integration of data from multiple studies increases the number of samples per condition and the total number of conditions, thereby adding power to the miRNA-mRNA association measure. Many of the microarray data sets in the Gene Expression Omnibus [31] repository correspond to either miRNA or mRNA expression. Consequently, the miRNA and mRNA data are unmatched and, even for a large number of biological conditions, the correlation coefficient cannot be used for measuring association. We present a novel association measure for analyzing unmatched data that correspond to a large number of conditions. In the next section, we apply the new association measures to two multiple myeloma (MM) data sets and identify several miRNA-mRNA pairs involved in apoptosis and cell proliferation.

## Results

We illustrate the applicability of the new association measures using two independently generated MM data sets. The first data set, henceforth referred to as the Lionetti data set [20], comprised healthy donors and MM patients stratified into five groups – TC1 to TC5 – based on TC [32] classification. The second data set, henceforth referred to as the Gutierrez data set [24], comprised healthy donors and MM patients with the following cytogenetic characteristics – normal FISH, t(11;14) (with or without RB deletion), t(4;14) (with or without RB deletion), and RB deletion as a unique abnormality. The two data sets were normalized as described in the Materials and Methods section. In the rest of this paper, RB deletion implies “RB deletion as a unique abnormality” and every MM group represents a biological condition of interest.

### Differentially expressed miRNAs and mRNAs

We considered a miRNA or mRNA to be DE in a biological condition of interest if the difference in average expression between the MM group and healthy individuals was statistically significant (refer Materials and Methods section).The Lionetti data set had five MM groups (i.e. TC1 to TC5) and the number of miRNAs and mRNAs that were DE in at least one of the five MM groups was 75 and 1024, respectively. Similarly, for the Gutierrez data set, the number of miRNAs and mRNAs that were DE in at least one of the four MM groups was 133 and 3486, respectively.

**Figure 1:**
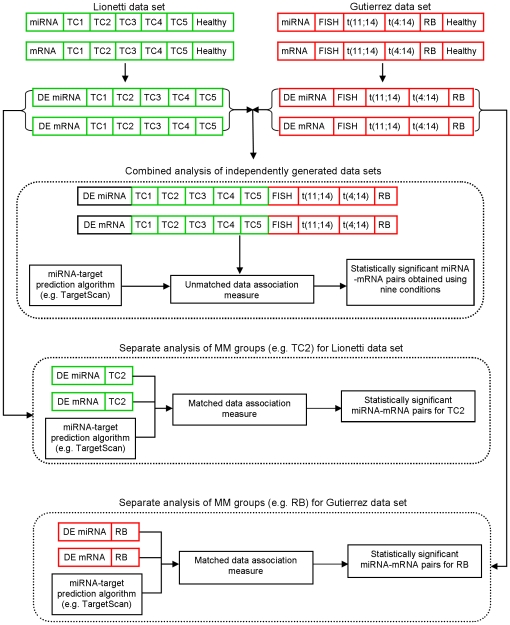
Identification of significant miRNA-mRNA pairs using association measures based on unmatched and matched data.

The correlation coefficient is an appropriate measure of association between miRNAs and mRNAs when the number of biological conditions is large. Since the Lionetti and Gutierrez data sets comprised fewer than six conditions, an identification of the significant miRNA-mRNA pairs using these data sets required the use of new association measures (Figure 1).

### Unmatched data association measure

The unmatched data (UD) association measure requires miRNA and mRNA expression profiles for several biological conditions and these conditions can be obtained by combining multiple data sets. We merged the Lionetti and Gutierrez data sets to obtain a “Master” data set comprising nine MM groups. The Master data set contained miRNAs and mRNAs that were DE in at least one of the groups (with respect to healthy individuals) and common to both Lionetti and Gutierrez data sets. Thus, we obtained a total of 120 miRNAs and 3260 mRNAs.

We use the notation “FC-value” to denote the difference between the average expression (miRNA or mRNA expression) in healthy donors and a MM group. We transformed the FC-values into +1, −1, or 0 using a discretization step (refer Materials and Methods section) and these transformed values are henceforth referred to as “discretized FC-values”. The discretized FC-value of −1 corresponds to an overexpression of miRNA/mRNA in healthy donors with respect to MM patients. Similarly, the discretized FC-value of +1 corresponds to an overexpression in MM patients with respect to healthy donors.

Once the discretized FC-values were obtained for the miRNAs and mRNAs in the Master data set, we determined the putative miRNA-mRNA pairs using TargetScanS. Although there are numerous miRNA-target prediction algorithms, they have similar sensitivity values [15] and we chose one of the commonly used algorithms for downstream analysis (refer Appendix S1 for an analysis based on miRBase). Of the 391,200 (120 miRNAs×3260 mRNAs) possible pairs, 6142 were predicted by TargetScanS. For each of the 6142 pairs, we used the discretized FC-values to test the null hypothesis that a change in mRNA expression is independent of a change in miRNA expression. We adjusted the *p*-values for multiple comparison using the Benjamini-Hochberg (BH) correction [33]. If the adjusted *p*-value was less than 0.05, then we rejected the null hypothesis and considered the association between the miRNA-mRNA pair to be of potential biological significance. We identified 40 of the 6142 pairs as significant and these pairs corresponded to 27 unique miRNAs and 18 unique mRNAs (Table S1).

**Figure 2:**
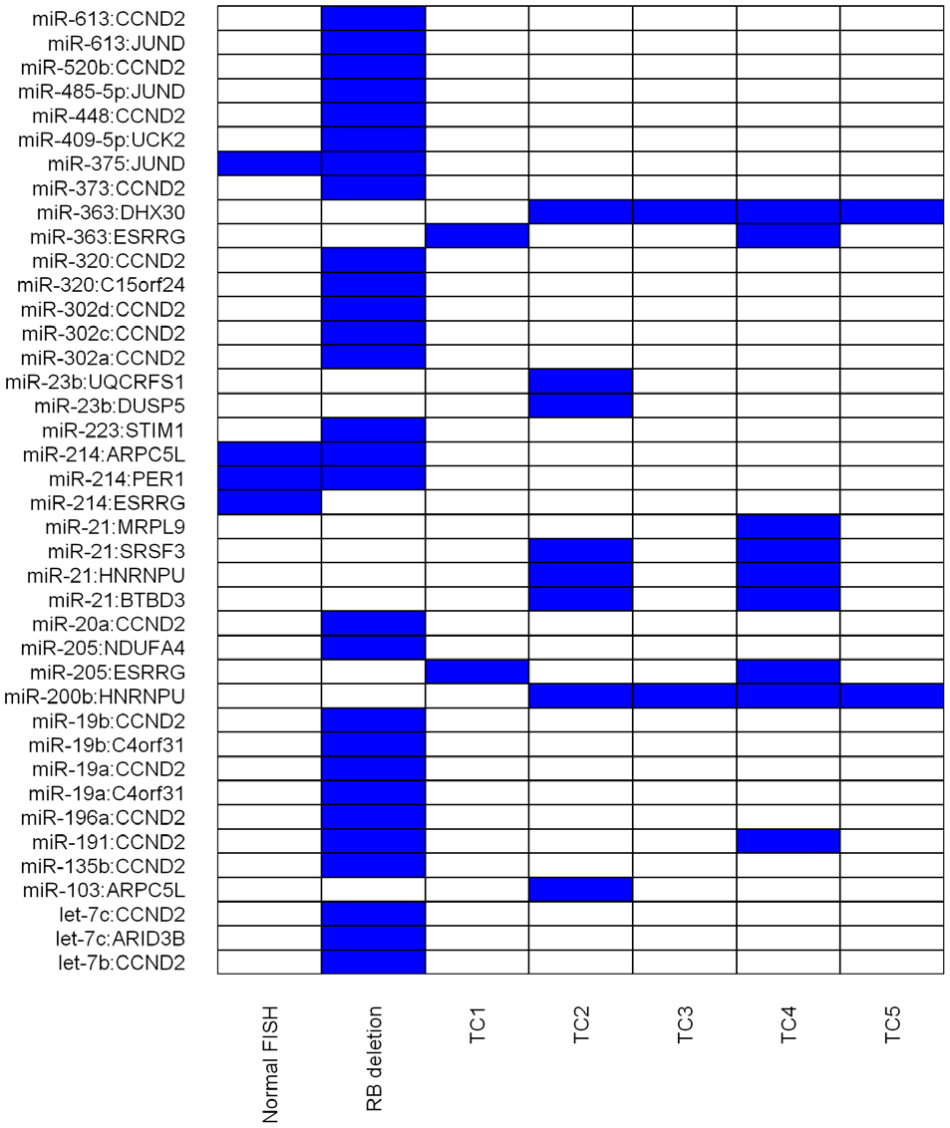
Significant miRNA-mRNA pairs obtained using unmatched data. The labels on the X-axis correspond to biological conditions and the labels on the Y-axis correspond to miRNA-mRNA pairs. Blue indicates that the miRNA-mRNA pair was statistically significant in the relevant condition.

We have previously shown using luciferase reporter assays that miRNAs can negatively regulate the translation of mRNA to protein [34] and similar results have been reported in other studies [9], [35]. Therefore, if the FC-value for a miRNA is positive (resp., negative), then the FC-value for the target mRNA must be negative (resp., positive). While the UD association measure can be used to identify miRNA-mRNA pairs with FC-values in the opposite direction or the same direction, in this paper we consider a pair to be significant only if the miRNA and mRNA FC-values are in the opposite direction for at least one of the biological conditions. In fact, for the master data set, some of the significant pairs had miRNA and mRNA FC-values in the opposite direction for more that one condition (Figure 2). For example, the pair hsa-miR-191:CCND2 had FC-values in the opposite direction for TC4 and RB deletion. Similarly, the pair hsa-miR-205:ESRRG had FC-values in the opposite direction for TC1 and TC4.

For a given condition, the calculation of miRNA and mRNA FC-values and, hence, the discretized FC-values, does not require the miRNA and mRNA expression to be obtained from the same set of individuals. Since the UD association measure only requires discretized FC-values as input, it is not dependent on the miRNA and mRNA data being matched.

### Matched data association measure

Instead of evaluating multiple MM groups to identify the significant miRNA-mRNA pairs, we could compare a MM group to healthy individuals and determine the miRNA-mRNA pairs of interest in the relevant MM group (Figure 1). Since the UD association measure is inappropriate for the latter analysis (refer to Appendix S2 for an explanation), we utilized the matched data (MD) association measure.

 The MD association measure considers a miRNA-mRNA pair to be of potential biological significance if the change in miRNA expression produces a change in mRNA expression in the opposite direction and the magnitude of change is higher than that by chance. The MD association measure requires the changes in miRNA and mRNA expression for the same set of individuals and is not applicable to unmatched data. Therefore, we analyzed the MM groups corresponding to Lionetti and Gutierrez data sets separately (Figure 1).

For the purpose of illustration, instead of analyzing all the nine MM groups, we focused on the two MM groups corresponding to the largest number of samples (or patients). For the Lionetti data set, the maximum number of samples was 10, which corresponded to patients with TC2 classification. For the Gutierrez data set, the maximum number of samples was 14, which corresponded to patients with RB deletion.

We use the notation “CE-value” to denote the change in miRNA/mRNA expression of a MM patient with respect to healthy donor. To identify the miRNA-mRNA pairs of potential biological significance in the RB deletion MM group, we first obtained the miRNA and mRNA CE-values for each of the 10 MM patients. Next, we provided these CE-values as input to the MD association measure and tested the null hypothesis that the change in mRNA expression is independent of a change in miRNA expression. We adjusted the *p*-values for multiple comparisons using the BH correction. If the adjusted *p*-value was less than 0.05 we rejected the null hypothesis and considered the miRNA-mRNA pair to be significant. Of the 6142 putative miRNA-mRNA pairs returned by TargetScanS, we identified 406 pairs as significant. These pairs corresponded to 47 unique miRNAs and 131 unique mRNAs (Table S2). Similarly, for an analysis involving TC2 patients, 187 of the 6142 pairs were observed to be significant. These pairs corresponded to 18 unique miRNAs and 85 unique mRNAs.

**Figure 3:**
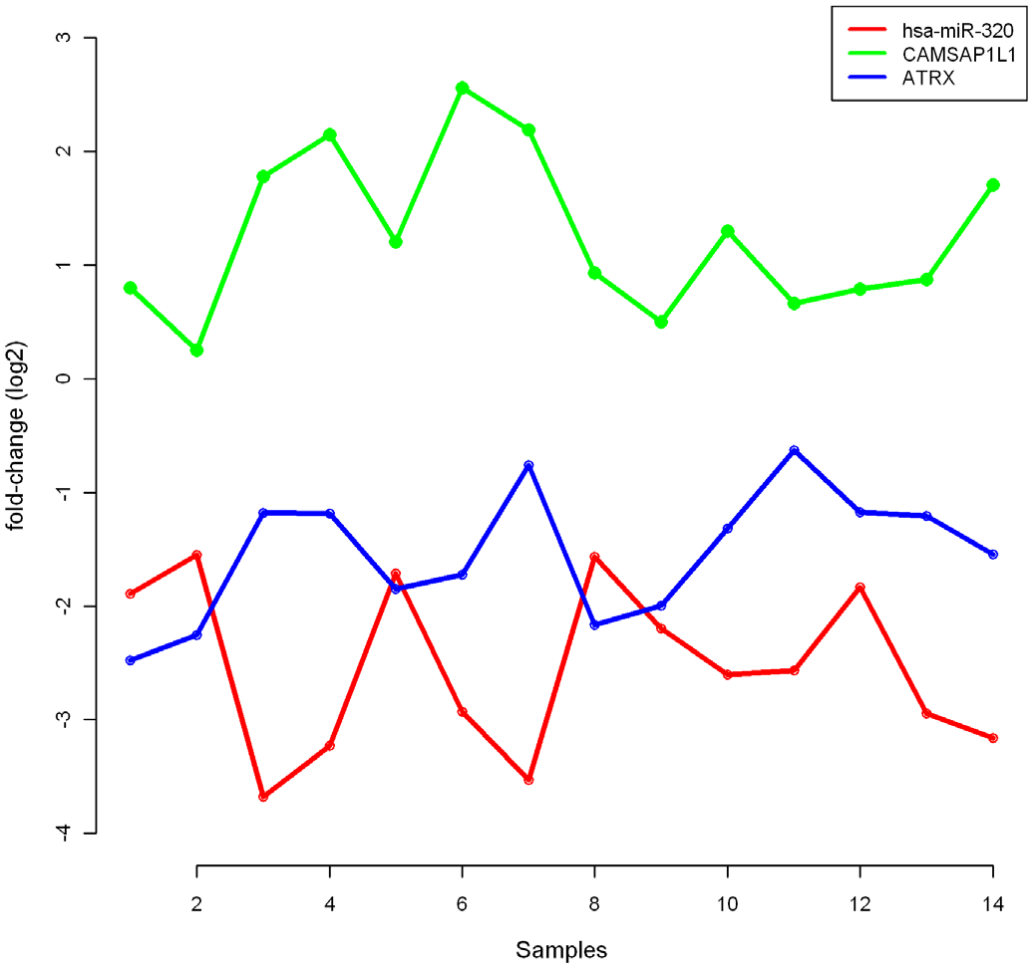
Relative expression levels of hsa-miR-320 and two of its predicted targets in samples with RB deletion.

 To compare the results obtained using the MD association measure with those obtained using correlation coefficient, we focused on patients with RB deletion. For every miRNA-mRNA pair that was predicted by TargetScanS, we used the miRNA and mRNA CE-values to obtain the correlation. Next, we performed a permutation test (refer Materials and Methods section) for the null hypothesis that the association between a miRNA and mRNA was not higher than that by chance. We adjusted the *p*-value for multiple comparisons using the BH correction and identified 19 of the 6142 miRNA-mRNA pairs as significant (adjusted *p*-value<0.05). The number of miRNA-mRNA pairs that were identified as significant using both correlation coefficient and MD association measure was just 3. For a miRNA-mRNA pair to be genuine, the average change in mRNA expression must be in the opposite direction to that of the miRNA. For example, if the average CE-value is positive for a miRNA, then the average CE-value must be negative for the target mRNA. Figure 3 shows the relationship between miRNA hsa-miR-320 and two of its putative targets, ATRX and CAMSAP1L1; the average CE-values were −2.53, −1.53, and 1.26, respectively.

Though the pair hsa-miR-320:ATRX was identified as significant using the correlation coefficient, the average CE-value was negative for both miRNA and mRNA. Therefore, ATRX cannot be considered a genuine target of hsa-miR-320. In contrast, the directions of average CE-values for hsa-miR-320 and CAMSAP1L1 were opposite, suggesting a potential targeting of CAMSAP1L1 by hsa-miR-320. In fact, the pair hsa-miR-320:CAMSAP1L1 was identified as significant using the correlation coefficient as well as the MD association measure.

### Concordance in results obtained using MD and UD association measures

While the MD association measure is suitable for analyzing one MM group at a time, the UD association measure is appropriate for analyzing several MM groups simultaneously. We analyzed nine MM groups using the UD association measure and two of these (RB deletion and TC2) were also analyzed using the MD association measure. For these two MM groups, we determined the overlap between the significant pairs identified using the two measures. As mentioned earlier, the UD association measure returned 40 significant pairs and each of these pairs corresponded to miRNA and mRNA FC-values in the opposite direction for at least one of the nine MM groups. We observed that 28 of the 40 pairs had RB deletion as one of the biological groups with FC-values in the opposite direction. The MD association measure returned 406 significant miRNA-mRNA pairs for RB deletion and these included the 28 pairs obtained using UD. Similarly, eight of the 40 significant pairs obtained using UD were associated with TC2 and these eight pairs were also identified using MD.

### Biological significance of miRNA-mRNA pairs

To illustrate the biological significance of the results obtained using UD and MD association measures, we focused on the MM group with the largest sample size, i.e. patients with RB deletion. Of the 406 significant miRNA-mRNA pairs obtained using the MD association measure, some were identified as significant in the Gutierrez study as well. These included hsa-miR-320a:MLLT3, hsa-miR-20a:CDKN1A, hsa-miR-20a:FURIN, hsa-miR-19b:IGF1, hsa-miR-15a:CCND2, hsa-miR-10b:KLF11, hsa-miR-19a:IGF1, and hsa-miR-19a:CCND2.

Among the miRNAs identified as significant many have previously been shown to be dysregulated in MM, e.g. hsa-miR-135b, -196a, -19a, -19b, -205, -214, -223, -320, -373, -375, -485-5p and -520b [24], [36]–[38]. Of significance is the identification of miR-19 family and in particular hsa-miR-19a and -19b; these two miRNAs have been linked to B cell neoplasms, including MM and have recently been directly implicated in MM pathogenesis [39], [40].

The 406 significant pairs corresponded to 131 unique mRNAs and a gene ontology (GO) analysis showed that many of these mRNAs were associated with apoptosis, cell proliferation and transcription regulation (Table 1). Overall, 57 miRNA-mRNA pairs were associated with apoptosis, 75 pairs were associated with transcription regulation, and 82 pairs were associated with cell proliferation (Table S3).

**Table 1:** Number of mRNAs associated with different biological processes in the RB deletion group.

Process	Number
Apoptosis	24
Signaling pathway	23
Transcription regulation	22
Cell proliferation	17
Cell cycle	8
Cell differentiation	5

**Figure 4:**
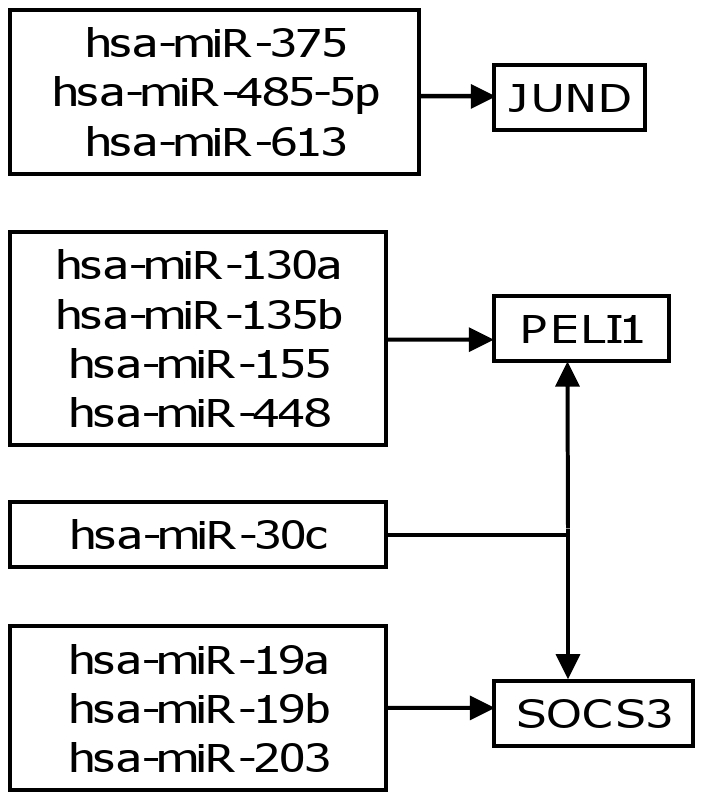
Network diagram comprising miRNAs that potentially regulate genes SOCS3, JUND, and PELI1.

Some of the genes of interest were SOCS3, JUND, and Pellino homolog 1 (PELI1) (Figure 4). Our MD association measure suggested that the anti-apoptotic gene SOCS3 was regulated by four miRNAs, including hsa-mir-19a and -19b. This gene is involved in the IL-6 signaling pathway which is important in myeloma cell survival [41]. The gene JUND has been shown to modulate the development of drug resistance in a majority of patients on Bortezomib-based anti-myeloma therapy [42]; three of the miRNAs identified in the original study as regulators of JUND were predicted using our MD model. The gene PELI1 has recently been shown to be involved in hsa-mir-21 mediated control of NF-κB signaling [43].

Among the 28 miRNA-mRNA pairs that were identified using both MD and UD, one of the genes of interest was cyclin D2 (CCND2). The 28 pairs corresponded to 22 unique miRNAs and 16 of these targeted CCND2. The cyclin D genes are regularly involved in chromosomal translocations in MM and the expression levels of these cyclins in myeloma tumors is extremely high in relation to normal proliferating peripheral blood cells [44]. A suppression of the expression of CCND2 using RNA interference (a method similar to miRNA inhibition) in myeloma cells inhibited proliferation and was progressively cytotoxic [45].

## Discussion

The new association measures enable the identification of miRNA-mRNA pairs of potential biological interest using microarray data sets that cannot be analyzed using the correlation coefficient (Table 2). Thus, our association measures extend the scope of data sets that can be used to generate new hypotheses and/or design new experiments.

**Table 2:** Association measures for co-analysis of miRNA and mRNA expression profiles.

		Number of conditions	
		Two	Large
**Expression profiles**	**Matched**	MD association measure	Correlation coefficient
	**Unmatched**	−	UD association measure

The MD association measure requires data for just two conditions and is suitable for analyzing pilot studies, where a small number of conditions are evaluated. The results obtained using the pilot studies can be used to design experiments for a comprehensive miRNA-mRNA analysis without having to effectively repeat the profiling experiments. Instead of a pilot study, one may utilize the previously published microarray profiling studies to identify the miRNA-mRNA pairs for further experimentation. However, many published studies comprise a small number of biological conditions (≤5) and do not include the expression levels of both miRNAs and mRNAs, e.g. [46]–[48]. The UD association measure, unlike the existing measures of association, can be used to co-analyze the independent miRNA and mRNA studies and identify the miRNA-mRNA pairs of potential interest. Thus, our UD and MD association measures allow researchers to obtain greater confidence in candidate miRNA-mRNA pairs before embarking on time-consuming and costly downstream experiments. For example, our analysis of the MM data sets supports a downstream experiment centered around the control of SOCS3 by the hsa-miR-19 family.

The discretization of expression data, prior to the calculation of UD-based association measure, enables us to focus on the directions of change in miRNA and mRNA expression (with respect to a reference) rather than the magnitudes of change. An advantage of this approach is that the discretized expression profiles are comparable across all microarray platforms. For example, the range of expression values obtained using Taqman low density arrays is very different from that measured using Agilent microarrays. Also, under different conditions the quantitative level of a miRNA's regulatory effect may vary. Therefore, if the actual expression values are used, then a UD-based method that adjusts for platform-specific and condition-specific differences will be needed; to the best of our knowledge, such methods are not available. The UD association measure is applicable to data sets where multiple conditions are evaluated. If the number of conditions is just two, then this association measure is inappropriate. However, there is no known solution for this limitation of the UD association measure.

While the UD association measure does not require miRNA and mRNA data from the same individual, the data must correspond to the same set of biological conditions. If there are multiple miRNA/mRNA data sets that correspond to the same set of biological conditions, then a representative data set may be selected prior to the estimation of association. This selection could be based on a measure of agreement, such as Cohen's kappa [49], between the data sets. Once all the pairwise agreements between the miRNA/mRNA data sets have been obtained, the data set with the highest average agreement can be selected as the representative data set.

Our measures of association can be used to identify miRNA-mRNA modules, i.e. clusters of miRNAs that regulate clusters of mRNAs. There are instances where two or more miRNAs collectively regulate multiple mRNAs and, in recent times, some methods have been proposed for obtaining such modules [27], [28], [30]. Since these module-identification methods utilize the correlation coefficient as the measure of association, they require matched miRNA-mRNA data for multiple conditions. Our UD association measure, unlike the correlation coefficient, enables an analysis of unmatched miRNA and mRNA data sets. Consequently, the UD association measure extends the scope of data sets that can be analyzed using the module-identification methods.

We showed that even when the miRNA and mRNA expression data are matched, the use of correlation coefficient may not be appropriate. Though the correlation coefficient is a measure of association between miRNA and mRNA expression, it is independent of the average CE-values. Consequently, the correlation coefficient may identify a miRNA-mRNA pair as significant even when the average CE-value is negative (or positive) for both miRNA and mRNA. In our analysis of MM patients with RB deletion, hsa-miR-320:ATRX was identified as significant using the correlation coefficient even though the average CE-value was negative for both miRNA and mRNA.

Our MD association measure assumes that if a miRNA targets an mRNA, then the change in mRNA expression due to a change in miRNA expression must be significant. However, there may be instances where several miRNAs cooperatively regulate an mRNA and the individual effect of a miRNA on mRNA expression is low. Our model can be extended to consider the coregulation of an mRNA by multiple miRNAs. Such a model may provide new insights into the miRNA-based regulatory mechanism.

## Materials and Methods

### Unmatched data

We first describe the method for identifying significant miRNA-mRNA pairs under the assumption that the miRNA and mRNA expression profiles are not matched. Even though we do not assume the expression profiles to be matched, we require the expression profiles to correspond to the same biological groups, e.g. the same MM groups. First, we select one of the biological groups in the data set, e.g. healthy donors, as the reference. Next, we test the null hypothesis that the average expression of a miRNA/mRNA in a MM group is the same as that for reference. If the *p*-value for the hypothesis, after adjusting for multiple comparisons, is less than 0.05, then we consider the miRNA/mRNA to be DE in the relevant MM group.

#### (i) Measure of association

For a given biological group, if the miRNA/mRNA is DE and overexpressed (with respect to reference), then it is assigned the value 1. Similarly, if the miRNA/mRNA is DE and underexpressed, then it is assigned the value −1. Finally, if the miRNA/mRNA is not DE, then it is assigned the value 0. We denote the total number of biological groups (excluding the reference) as C. Therefore, for every miRNA/mRNA, we obtain a vector of C discrete values. The discretized data are used to populate the 3×3 contingency table (Table 3) shown below.

**Table 3:** Generic table for measuring association between a miRNA-mRNA pair using unmatched data.

			mRNA	
		−1	0	1
**miRNA**	**−1**	a_11_	a_12_	a_13_
	**0**	a_21_	a_22_	a_23_
	**1**	a_31_	a_32_	a_33_

Here, a_11_ corresponds to the number of biological groups where both miRNA and mRNA are underexpressed with respect to reference. We use α and β to denote the miRNA and mRNA of interest, respectively, and α:β to denote the miRNA-mRNA pair. Therefore, for miRNA α and mRNA β, 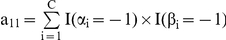, where I is an indicator function that takes the values 1 if the condition is satisfied and 0, otherwise. Similarly, we obtain a_12_–a_33_.

#### (ii) Significance of association

Once the 3×3 contingency table has been populated for a given miRNA-mRNA pair, we calculate the probability of obtaining the observed set of nine values (a_11_,…,a_33_) by chance. To determine this probability, we assume that the nine values are obtained from a multinomial distribution and that there is no biological association between α and β.

Given that every mRNA has C discrete values (corresponding to the C biological groups), firstly, we obtain a matrix **W**_1_ comprising Z rows and C columns, where Z denotes the number of mRNAs. Secondly, we jumble the rows and columns of **W**_1_ to obtain a new matrix **W**_2_. Specifically, each row of **W**_2_ has C values and these are selected at random from the Z×C values in **W**_1_. Thirdly, we sample with replacement the rows from matrix **W**_2_ and obtain the matrix **W**_3_. The number of times the sampling is performed, i.e. N_rep_, is user-defined and, in this paper, all results were obtained using N_rep_ = 10000. The matrix **W**_3_ comprises 10000 pseudo-mRNAs that have no genuine association to the miRNA α. Finally, we populate the 3×3 contingency table (Table 2) using the discretized values for miRNA α and the N_rep_ pseudo-mRNAs. We obtain the average values for the 9 elements a_11_,…,a_33_ by dividing the values in the contingency table by N_rep_. These average values represent the probabilities for the nine elements under the assumption that there is no biological association between the miRNA α and an mRNA. We provide these probabilities as input to the R [50] package EMT and determine the *p*-value, for the observed association between miRNA α and its putative target β, using a multinomial distribution. Instead of a test based on multinomial distribution, a χ^2^-test can be performed if the number of conditions is large (>15).

#### (iii) miRNA-mRNA pairs of interest

A miRNA α is predicted to target several mRNAs and the test of significance described above has to be performed for all the targets of α. Since miRNAs are negative regulators of mRNA expression, the change in the expression of a genuine target must be in the opposite direction to that for the miRNA. Therefore, instead of evaluating all the predicted targets of α, we only consider those targets which have an opposite direction of change (with respect to α) in at least one of the C biological groups. We adjust the *p*-values (returned by the test of significance) for multiple comparisons using the Benjamini-Hochberg (BH) correction [33]. We consider a miRNA-mRNA pair to be significant if the adjusted *p*-value is less than 0.05.

### Matched data

We now describe the method for the identification of significant miRNA-mRNA pairs using expression data that are matched but correspond to just two conditions. Firstly, we select the samples corresponding to one of the conditions as the reference, e.g. in an analysis involving a MM group and healthy donors, the latter may be selected as the reference. Secondly, we model the change in mRNA expression as a function of the change in miRNA expression and obtain the statistical significance of association for the miRNA-mRNA pair. The actual steps are described in detail below:

#### (i) Measure of associatio

Let the number of healthy donors and MM samples be N_ref_ and N_biol_, respectively. Typically, N_ref_≪N_biol_, e.g. the Gutierrez data set had 14 MM samples with RB deletion and just three healthy donors, and our measure of association focuses on such cases. Let μ and υ denote the median mRNA and miRNA expression values, respectively, for the healthy donors. We consider the median values instead of average values because the former are not sensitive to the presence of outliers. Let λ denote the change in mRNA expression owing to a unit change in miRNA expression. Let x_i_ and y_i_ denote the miRNA and mRNA expressions, respectively, for the i^th^ MM sample. Now, we model the relationship between miRNA and mRNA expression in MM samples as follows:
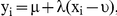
where 1≤i≤N_biol_

or
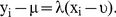
(1)For two-channel experiments, every microarray slide contains the expression profile of a MM sample and healthy donor. Therefore, we directly obtain the CE-values, i.e. y_i_−μ for mRNAs and x_i_−υ for miRNAs. In contrast, for single-channel experiments, a microarray slide corresponds to the expression profile of a healthy donor or MM sample and the CE-values have to be obtained explicitly.

#### (ii) Significance of association

Once the value of λ has been estimated using Equation (1), the next step is to ascertain its statistical significance. Specifically, if a miRNA-mRNA pair is genuine, then the change in miRNA expression must produce a change in mRNA expression which is higher than that obtained by chance. We achieve this by performing a permutation test similar to that described earlier for the identification of significant miRNA-mRNA pairs using unmatched data.

Let **Q**_1_ denote the matrix of Z rows (corresponding to Z mRNAs) and N_biol_ columns. Next, we jumble the rows and columns of **Q**_1_, in a manner similar to that described for UD, and obtain a new matrix **Q**_2_. We sample with replacement the rows from matrix **Q**_2_ and obtain the matrix **Q**_3_. The sampling is performed N_rep_ times and each sample represents a pseudo-mRNA with no genuine association to miRNA α.

We use Equation (1) to estimate λ for the association between miRNA α and every pseudo-mRNA in **Q**_3_. These values of λ represent the null distribution, i.e. the distribution when miRNA α and an mRNA do not have a biological association. If the α:β pair is biologically important, then the observed value of λ (i.e. λ^obs^) must be negative (implying a negative regulation of the mRNA by α) and smaller than the values in the null distribution. Therefore, the *p*-value is obtained as Pr(λ≤λ^obs^).

#### (iii) miRNA-mRNA pairs of interest

For a given miRNA α, we first identify all the predicted targets that have an average CE-value in the opposite direction to that for the miRNA. Next, we obtain the *p*-values using the test of significance described above. Finally, we adjust the *p*-values for multiple comparisons using the BH correction. We consider a miRNA-mRNA pair to be significant if λ^obs^ is negative and the adjusted *p*-value is less than 0.05.

### Biological data sets

We considered two publicly available data sets – Gutierrez data set [24] and Lionetti data set [20]. The Gutierrez data set comprised five healthy donors and 55 MM patients that were stratified into four groups – normal FISH, t(11:14), t(4;14) and RB deletion. The raw miRNA and mRNA expression values were downloaded from Gene Expresssion Omnibus (GEO) accession number GSE16558. The miRNA expression profiles were obtained using TaqMan low-density arrays and normalized using the mean of RNU44 and RNU48, as suggested by the authors [24]. The mRNA expression profiles were obtained using Affymetrix Human Gene 1.0 ST arrays and the preprocessing steps included RMA background correction [51], quantile normalization [52] and summarization of mRNA expression using the median polish algorithm. The Lionetti data set contained 40 MM patients (stratified into five groups based on TC classification), three healthy donors for miRNAs, and four healthy donors for mRNAs. The normalized miRNA and mRNA expression values (Figure S1) were downloaded from GEO accession numbers GSE17498 and GSE13591, respectively.

For each data set, we tested the null hypothesis that the average expression of a miRNA/mRNA in a MM group was the same as that for healthy donors. We adjusted the *p*-values (corresponding to the tests of hypotheses) for multiple comparisons using the BH method. We considered a miRNA/mRNA to be DE if the difference in average expression was greater than 1.5 and the adjusted *p*-value was less than 0.05.

## Supporting Information

Figure S1 Boxplots of normalized (a) miRNA expression values and (b) mRNA expression values for Lionetti data set.
(TIF)

Table S1 Significant miRNA-mRNA pairs obtained using TargetScanS for unmatched data.
(DOC)

Table S2 Significant miRNA-mRNA pairs obtained using TargetScanS for the RB deletion MM group.
(DOC)

Table S3 miRNA-mRNA pairs associated with apoptosis, transcription regulation, and cell proliferation in the RB deletion group.
(DOC)

Appendix S1 Analysis of unmatched miRNA-mRNA data using miRBase.
(DOC)

Appendix S2 An illustration of the results obtained using UD association measure as the number of conditions varies.
(DOC)
