# Supplementary figures and images for: Measures of Association for Identifying MicroRNA-mRNA Pairs of Biological Interest
Source: PLoS One. 2012 Jan 11;7(1):e29612. doi: 10.1371/journal.pone.0029612 (PMC3256172; doi:10.1371/journal.pone.0029612)

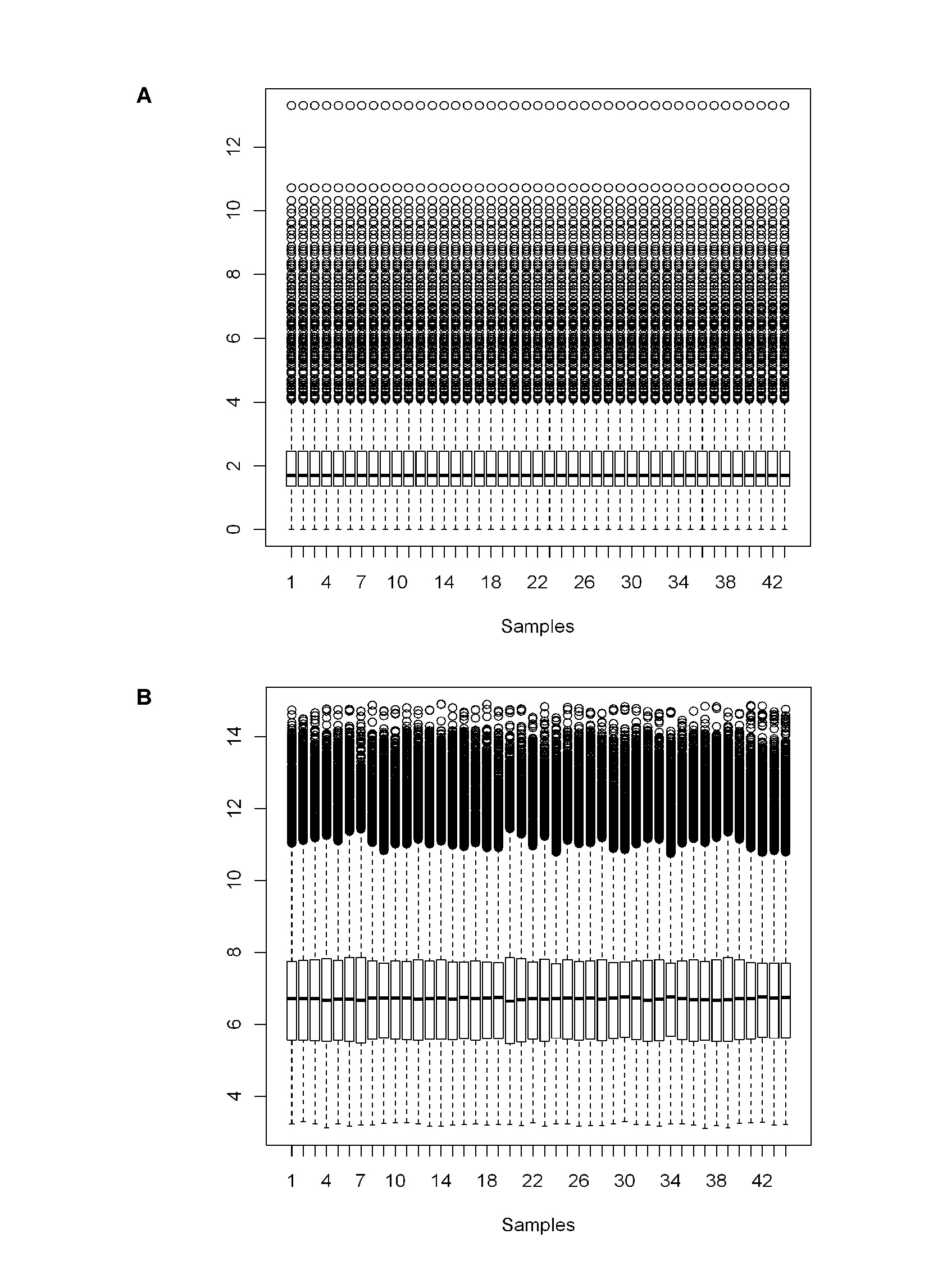

Supplement: Figure S1 — Boxplots of normalized (a) miRNA expression values and (b) mRNA expression values for Lionetti data set. (TIF) [file pone.0029612.s001.tif]
