# Supplementary material for: Measures of Association for Identifying MicroRNA-mRNA Pairs of Biological Interest
Source: PLoS One. 2012 Jan 11;7(1):e29612. doi: 10.1371/journal.pone.0029612 (PMC3256172; doi:10.1371/journal.pone.0029612)
Supplement: Table S1 — Significant miRNA-mRNA pairs obtained using TargetScanS for unmatched data. (DOC) [file pone.0029612.s002.doc]

| **miRNA** | **Potential targets** |
| --- | --- |
| hsa-miR-205 | ESRRG, NDUFA4 |
| hsa-miR-20a | CCND2 |
| hsa-miR-21 | BTBD3, HNRNPU, SRSF3, MRPL9 |
| hsa-miR-214 | ESRRG, PER1, ARPC5L |
| hsa-miR-223 | STIM1 |
| hsa-miR-23b | DUSP5, UQCRFS1 |
| hsa-miR-302a | CCND2 |
| hsa-miR-302c | CCND2 |
| hsa-miR-302d | CCND2 |
| hsa-miR-320 | C15orf24, CCND2 |
| hsa-miR-363 | ESRRG, DHX30 |
| hsa-miR-373 | CCND2 |
| hsa-miR-375 | JUND |
| hsa-miR-409-5p | UCK2 |
| hsa-miR-448 | CCND2 |
| hsa-miR-485-5p | JUND |
| hsa-miR-520b | CCND2 |
| hsa-miR-613 | JUND, CCND2 |
