# Supplementary material for: Measures of Association for Identifying MicroRNA-mRNA Pairs of Biological Interest
Source: PLoS One. 2012 Jan 11;7(1):e29612. doi: 10.1371/journal.pone.0029612 (PMC3256172; doi:10.1371/journal.pone.0029612)
Supplement: Table S2 — Significant miRNA-mRNA pairs obtained using TargetScanS for the RB deletion MM group. (DOC) [file pone.0029612.s003.doc]

| **miRNA** | **Potential targets** |
| --- | --- |
| hsa-let-7b | TRIB1, ARID3B, PIK3IP1, RBFOX2, GNAL, GNG5, SLC25A4, PBX2, YOD1, GPR137, SLCO5A1, SLC5A6, CCND2, RNF7, SPATA2, POM121 |
| hsa-let-7c | TRIB1, ARID3B, PIK3IP1, RBFOX2, GNAL, GNG5, SLC25A4, PBX2, YOD1, GPR137, SLCO5A1, SLC5A6, CCND2, RNF7, SPATA2, POM121 |
| hsa-miR-10a | YOD1, BACH2, USF2, KLF11, TMEM183A |
| hsa-miR-10b | YOD1, BACH2, USF2, KLF11, TMEM183A |
| hsa-miR-130a | PIK3IP1, GADD45A, SHANK2, RBFOX2, CCRN4L, ITPK1, LDLR, IER3IP1, PELI1, BACH2, SKP1, C18orf1, VPS37B, SPATA2 |
| hsa-miR-133b | SF3B4, SEC61B, PLK3, COX6A1, GABARAPL1, ARHGDIA, MLLT3, SGK1, TPI1, TAGLN2, CD47, CDC42 |
| hsa-miR-135b | NAMPT, DAG1, DMWD, RBFOX2, ORMDL2, PELI1, TWF1, FMNL1, ARID5B, CCND2, CD47, TRAF4, SPATA2 |
| hsa-miR-155 | NAMPT, EHD1, KAT2A, PELI1, SMARCA4, CD47 |
| hsa-miR-15a | KIF1C, ENSA, TRAK1, PPRC1, KLHL18, PHF15, RBFOX2, KDSR, ARHGDIA, SMAD7, FURIN, UBAP1, PIM1, PI4KB, YOD1, BACH2, BCL9, SGK1, CCDC6, CCND2, DEDD, POM121 |
| hsa-miR-186 | SHANK2, CAMSAP1L1, PHF15, SRGAP2, RBFOX2, IGF1, MLLT3, OAZ2, BACH2, BCL9, USF2, NCK2, CCND2, CDC42 |
| hsa-miR-191 | CCND2 |
| hsa-miR-196a | YOD1, CCND2 |
| hsa-miR-19a | DAG1, SHANK2, RBFOX2, CCRN4L, IGF1, ASNA1, PPPDE1, FEM1C, BCL3, SGK1, C4orf31, VPS37B, ARID5B, CCND2, SOCS3, SPATA2 |
| hsa-miR-19b | DAG1, SHANK2, RBFOX2, CCRN4L, IGF1, ASNA1, PPPDE1, FEM1C, BCL3, SGK1, C4orf31, VPS37B, ARID5B, CCND2, SOCS3, SPATA2 |
| hsa-miR-203 | EDN1, CAMSAP1L1, GABARAPL1, ABHD14A, TWF1, SOCS3, CD47 |
| hsa-miR-205 | CAMSAP1L1, RBFOX2, RND3, LMNA, NDUFA4, PRKCE, KLF11 |
| hsa-miR-20a | CDKN1A, DUSP8, KPNA2, LDLR, SMAD7, MAP3K11, FURIN, HN1, PLXNA1, YOD1, FEM1C, TWF1, RRAGD, EIF4H, KLF11, SQSTM1, CCND2, DEDD |
| hsa-miR-214 | CIB1, PHF15, FAM53C, PER1, PIM1, CDC42SE1, TWF1, ARPC5L, HMGN3, CD47 |
| hsa-miR-217 | FEM1C |
| hsa-miR-221 | HNRNPA0, SHANK2, KPNA2 |
| hsa-miR-223 | DUSP10, DAG1, SLC39A1, STIM1 |
| hsa-miR-224 | RND3, YOD1, CDC42 |
| hsa-miR-24 | PLK3, FURIN, PER1, PIM1, YOD1, ARID5B |
| hsa-miR-299-3p | CHST2, CDC42 |
| hsa-miR-302a | KLHL18, KPNA2, MAP3K11, HN1, PLXNA1, YOD1, FEM1C, TWF1, RRAGD, RDBP, CCND2 |
| hsa-miR-302b | KLHL18, KPNA2, MAP3K11, HN1, PLXNA1, YOD1, FEM1C, TWF1, RRAGD, RDBP, CCND2 |
| hsa-miR-302c | KLHL18, KPNA2, MAP3K11, HN1, PLXNA1, YOD1, FEM1C, TWF1, RRAGD, RDBP, CCND2 |
| hsa-miR-302d | KLHL18, KPNA2, MAP3K11, HN1, PLXNA1, YOD1, FEM1C, TWF1, RRAGD, RDBP, CCND2 |
| hsa-miR-30c | DAG1, PHF15, IL1A, ITPK1, RND3, PPPDE1, IER5, PLXNA1, SEC61A2, YOD1, C9orf86, PELI1, TWF1, BACH2, BCL9, VIM, ARID5B, NCK2, KLF11, SOCS3, CHST2 |
| hsa-miR-320 | PLK3, DAG1, SPRED2, CAMSAP1L1, IGF1, RHOG, MLLT3, YOD1, C15orf24, TWF1, VPS37B, ARID5B, CCND2 |
| hsa-miR-325 | PLXNA1, CCDC6 |
| hsa-miR-329 | CLTB, RNF19B, SPRED2, BCL9, ARID5B, PEA15 |
| hsa-miR-363 | PRKCE, BACH2, CCDC6 |
| hsa-miR-367 | HNRNPA0, DUSP10, DAG1, KLHL18, PHF15, RBFOX2, NELF, MKNK2, SMAD7, PRKCE, BCL9, SGK1, USF2, C18orf1, HPS6, NCK2, CDC42 |
| hsa-miR-373 | KLHL18, KPNA2, MAP3K11, HN1, PLXNA1, YOD1, FEM1C, TWF1, RRAGD, RDBP, CCND2 |
| hsa-miR-375 | ORMDL2, JUND, CCDC6 |
| hsa-miR-376b | HNRNPA0 |
| hsa-miR-409-5p | RND3, SAMD4B, UCK2 |
| hsa-miR-429 | DAG1, CAMSAP1L1, RBFOX2, RND3, ARHGDIA, IER5, BCL9, TCEB1, TUBB2A, NCK2, CHST2 |
| hsa-miR-448 | SBNO2, KDSR, TOR1AIP1, MKNK2, FURIN, PPPDE1, RTN4, PELI1, BACH2, C18orf1, SLCO5A1, CCND2 |
| hsa-miR-451 | SAMD4B |
| hsa-miR-485-5p | DAG1, CBX6, KDSR, JUND, MLLT3, FAM53C, RDBP |
| hsa-miR-505 | SRGAP2, CBX6 |
| hsa-miR-520b | KLHL18, KPNA2, MAP3K11, HN1, PLXNA1, YOD1, FEM1C, TWF1, RRAGD, RDBP, CCND2 |
| hsa-miR-544 | GNAL, GTF2A2, H2AFZ, PEA15 |
| hsa-miR-613 | NAMPT, EDN1, SRGAP2, IGF1, JUND, MLLT3, PIM1, TWF1, BACH2, SMARCA4, TAGLN2, NCK2, CCND2, CDC42 |
| hsa-miR-99b | TRIB1 |
