# Supplementary material for: Measures of Association for Identifying MicroRNA-mRNA Pairs of Biological Interest
Source: PLoS One. 2012 Jan 11;7(1):e29612. doi: 10.1371/journal.pone.0029612 (PMC3256172; doi:10.1371/journal.pone.0029612)
Supplement: Table S3 — miRNA-mRNA pairs associated with apoptosis, transcription regulation, and cell proliferation in the RB deletion group. (DOC) [file pone.0029612.s004.doc]

| **Term** | **miRNA-mRNA pairs** |
| --- | --- |
| Apoptosis | hsa-let-7b:RNF7, hsa-let-7c:RNF7, hsa-miR-10a:KLF11, hsa-miR-10b:KLF11, hsa-miR-130a:GADD45A, hsa-miR-133b:ARHGDIA, hsa-miR-133b:SGK1, hsa-miR-135b:TRAF4, hsa-miR-155:KAT2A, hsa-miR-15a:ARHGDIA, hsa-miR-15a:SMAD7, hsa-miR-15a:PIM1, hsa-miR-15a:SGK1, hsa-miR-15a:DEDD, hsa-miR-186:IGF1, hsa-miR-19a:IGF1, hsa-miR-19a:BCL3, hsa-miR-19a:SGK1, hsa-miR-19a:SOCS3, hsa-miR-19b:IGF1, hsa-miR-19b:BCL3, hsa-miR-19b:SGK1, hsa-miR-19b:SOCS3, hsa-miR-203:SOCS3, hsa-miR-205:LMNA, hsa-miR-205:PRKCE, hsa-miR-205:KLF11, hsa-miR-20a:CDKN1A, hsa-miR-20a:SMAD7, hsa-miR-20a:MAP3K11, hsa-miR-20a:KLF11, hsa-miR-20a:SQSTM1, hsa-miR-20a:DEDD, hsa-miR-214:CIB1, hsa-miR-214:PIM1, hsa-miR-24:PIM1, hsa-miR-302a:MAP3K11, hsa-miR-302b:MAP3K11, hsa-miR-302c:MAP3K11, hsa-miR-302d:MAP3K11, hsa-miR-30c:IL1A, hsa-miR-30c:VIM, hsa-miR-30c:KLF11, hsa-miR-30c:SOCS3, hsa-miR-320:IGF1, hsa-miR-329:PEA15, hsa-miR-363:PRKCE, hsa-miR-367:SMAD7, hsa-miR-367:PRKCE, hsa-miR-367:SGK1, hsa-miR-373:MAP3K11, hsa-miR-429:ARHGDIA, hsa-miR-448:RTN4, hsa-miR-520b:MAP3K11, hsa-miR-544:PEA15, hsa-miR-613:IGF1, hsa-miR-613:PIM1 |
| Transcription regulation | hsa-let-7b:ARID3B, hsa-let-7b:RBFOX2, hsa-let-7b:PBX2, hsa-let-7c:ARID3B, hsa-let-7c:RBFOX2, hsa-let-7c:PBX2, hsa-miR-10a:BACH2, hsa-miR-10a:USF2, hsa-miR-10a:KLF11, hsa-miR-10b:BACH2, hsa-miR-10b:USF2, hsa-miR-10b:KLF11, hsa-miR-130a:RBFOX2, hsa-miR-130a:BACH2, hsa-miR-133b:MLLT3, hsa-miR-135b:RBFOX2, hsa-miR-135b:ARID5B, hsa-miR-155:KAT2A, hsa-miR-15a:TRAK1, hsa-miR-15a:PPRC1, hsa-miR-15a:RBFOX2, hsa-miR-15a:BACH2, hsa-miR-15a:DEDD, hsa-miR-186:RBFOX2, hsa-miR-186:IGF1, hsa-miR-186:MLLT3, hsa-miR-186:BACH2, hsa-miR-186:USF2, hsa-miR-19a:RBFOX2, hsa-miR-19a:IGF1, hsa-miR-19a:BCL3, hsa-miR-19a:ARID5B, hsa-miR-19b:RBFOX2, hsa-miR-19b:IGF1, hsa-miR-19b:BCL3, hsa-miR-19b:ARID5B, hsa-miR-205:RBFOX2, hsa-miR-205:KLF11, hsa-miR-20a:KLF11, hsa-miR-20a:SQSTM1, hsa-miR-20a:DEDD, hsa-miR-214:PER1, hsa-miR-24:PER1, hsa-miR-24:ARID5B, hsa-miR-302a:RDBP, hsa-miR-302b:RDBP, hsa-miR-302c:RDBP, hsa-miR-302d:RDBP, hsa-miR-30c:BACH2, hsa-miR-30c:ARID5B, hsa-miR-30c:KLF11, hsa-miR-320:IGF1, hsa-miR-320:RHOG, hsa-miR-320:MLLT3, hsa-miR-320:ARID5B, hsa-miR-329:ARID5B, hsa-miR-363:BACH2, hsa-miR-367:RBFOX2, hsa-miR-367:USF2, hsa-miR-373:RDBP, hsa-miR-375:JUND, hsa-miR-429:RBFOX2, hsa-miR-429:TCEB1, hsa-miR-448:SBNO2, hsa-miR-448:BACH2, hsa-miR-485-5p:CBX6, hsa-miR-485-5p:JUND, hsa-miR-485-5p:MLLT3, hsa-miR-485-5p:RDBP, hsa-miR-505:CBX6, hsa-miR-520b:RDBP, hsa-miR-613:IGF1, hsa-miR-613:JUND, hsa-miR-613:MLLT3, hsa-miR-613:BACH2 |
| Cell Proliferation | hsa-let-7b:TRIB1, hsa-let-7b:RBFOX2, hsa-let-7b:CCND2, hsa-let-7c:TRIB1, hsa-let-7c:RBFOX2, hsa-let-7c:CCND2, hsa-miR-10a:KLF11, hsa-miR-10b:KLF11, hsa-miR-130a:RBFOX2, hsa-miR-130a:PELI1, hsa-miR-133b:CD47, hsa-miR-135b:NAMPT, hsa-miR-135b:RBFOX2, hsa-miR-135b:PELI1, hsa-miR-135b:CCND2, hsa-miR-135b:CD47, hsa-miR-155:NAMPT, hsa-miR-155:KAT2A, hsa-miR-155:PELI1, hsa-miR-155:CD47, hsa-miR-15a:RBFOX2, hsa-miR-15a:FURIN, hsa-miR-15a:PIM1, hsa-miR-15a:CCND2, hsa-miR-186:RBFOX2, hsa-miR-186:IGF1, hsa-miR-186:NCK2, hsa-miR-186:CCND2, hsa-miR-191:CCND2, hsa-miR-196a:CCND2, hsa-miR-19a:RBFOX2, hsa-miR-19a:IGF1, hsa-miR-19a:CCND2, hsa-miR-19b:RBFOX2, hsa-miR-19b:IGF1, hsa-miR-19b:CCND2, hsa-miR-203:EDN1, hsa-miR-203:CD47, hsa-miR-205:RBFOX2, hsa-miR-205:KLF11, hsa-miR-20a:CDKN1A, hsa-miR-20a:MAP3K11, hsa-miR-20a:FURIN, hsa-miR-20a:KLF11, hsa-miR-20a:CCND2, hsa-miR-214:PIM1, hsa-miR-214:CD47, hsa-miR-24:FURIN, hsa-miR-24:PIM1, hsa-miR-302a:MAP3K11, hsa-miR-302a:CCND2, hsa-miR-302b:MAP3K11, hsa-miR-302b:CCND2, hsa-miR-302c:MAP3K11, hsa-miR-302c:CCND2, hsa-miR-302d:MAP3K11, hsa-miR-302d:CCND2, hsa-miR-30c:IL1A, hsa-miR-30c:PELI1, hsa-miR-30c:NCK2, hsa-miR-30c:KLF11, hsa-miR-320:IGF1, hsa-miR-320:RHOG, hsa-miR-320:CCND2, hsa-miR-367:RBFOX2, hsa-miR-367:NCK2, hsa-miR-373:MAP3K11, hsa-miR-373:CCND2, hsa-miR-429:RBFOX2, hsa-miR-429:NCK2, hsa-miR-448:FURIN, hsa-miR-448:PELI1, hsa-miR-448:CCND2, hsa-miR-520b:MAP3K11, hsa-miR-520b:CCND2, hsa-miR-613:NAMPT, hsa-miR-613:EDN1, hsa-miR-613:IGF1, hsa-miR-613:PIM1, hsa-miR-613:NCK2, hsa-miR-613:CCND2, hsa-miR-99b:TRIB1 |
