# Supplementary material for: Measures of Association for Identifying MicroRNA-mRNA Pairs of Biological Interest
Source: PLoS One. 2012 Jan 11;7(1):e29612. doi: 10.1371/journal.pone.0029612 (PMC3256172; doi:10.1371/journal.pone.0029612)
Supplement: Appendix S1 — Analysis of unmatched miRNA-mRNA data using miRBase. (DOC) [file pone.0029612.s005.doc]

For the unmatched data set comprising nine biological conditions, we calculated the discretized expression values for 120 miRNAs and 3260 mRNAs. We observed that 15532 of the 391200 (120×3260) possible pairs were predicted by miRBase. Of the 15532 putative miRNA-mRNA pairs, 274 were found to be significant with a *p*-value less than 0.05. These corresponded to 95 unique miRNAs and 78 unique mRNAs (Table 1).

Table 1: Significant miRNA-mRNA pairs obtained using miRBase

| **miRNA** | **Potential Targets** |
| --- | --- |
| hsa-let-7c | HIGD2A, NME1, C19orf56 |
| hsa-miR-1 | PLA2G7, CD19 |
| hsa-miR-10a | ARID5A, CLIC1, HIST1H2AC |
| hsa-miR-10b | ARID5A, HIST1H2AC |
| hsa-miR-130a | NDUFA2, NME1, SNRPE |
| hsa-miR-133a | PLA2G7 |
| hsa-miR-133b | CLIC1, NME1, PLA2G7 |
| hsa-miR-141 | RANBP6, NME1, RPLP1, SIAH2, C4orf31 |
| hsa-miR-147 | SNRPE |
| hsa-miR-148a | ERCC5, OPHN1, VCAM1 |
| hsa-miR-155 | LDHA, RPLP1 |
| hsa-miR-15a | HIGD2A, C19orf56 |
| hsa-miR-186 | COX7A2, NME1, RPS10 |
| hsa-miR-18a | GAPDH, C20orf24 |
| hsa-miR-196a | IFRD2 |
| hsa-miR-19a | C15orf24 |
| hsa-miR-19b | C15orf24 |
| hsa-miR-200a | NME1, PSMB7, RPLP1 |
| hsa-miR-200b | EIF4A1, RANBP6, HSPA9, ATP5B, SNRPE, IFRD2 |
| hsa-miR-200c | EIF4A1, ATP5B, SNRPE, IFRD2 |
| hsa-miR-202 | CLIC1, HIGD2A, IFRD2 |
| hsa-miR-203 | CLIC1, DUSP5, C20orf24 |
| hsa-miR-205 | CLIC1, DDT, RPL36, EHD4, C19orf56, TMEM111, RPS18 |
| hsa-miR-20a | TUBB2C |
| hsa-miR-214 | TUBB2C, RPL36, NDUFV1, C19orf56, RPS10, IFRD2, ARPC5L |
| hsa-miR-221 | NDUFA2, IFRD2 |
| hsa-miR-222 | PSMB7 |
| hsa-miR-223 | PPP1R14B, GPI, HIST1H1C, C4orf31, ARPC5L |
| hsa-miR-23b | DUSP5, NDUFA2, RPLP1, UQCRFS1 |
| hsa-miR-24 | ARID5A |
| hsa-miR-299-3p | TUBB2C, GPI, ARHGAP4, RPS19, RPS24 |
| hsa-miR-302a | CLIC1 |
| hsa-miR-302b | TUBB2C, CLIC1, SNRPE |
| hsa-miR-302c | TUBB2C, CLIC1, SNRPE |
| hsa-miR-302d | TUBB2C, CLIC1 |
| hsa-miR-30c | LDHA, RPS19, C4orf31 |
| hsa-miR-320 | SCAMP3, RPL35, COX7C, DDT, PPP1R14B, ARHGAP4, C15orf24, NR1H2, IFRD2 |
| hsa-miR-325 | CLIC1, NDUFA2, UCK2 |
| hsa-miR-329 | RPL36, NME1, RPLP1 |
| hsa-miR-363 | DUSP10, DBI, DUSP5, NDUFA2 |
| hsa-miR-365 | IRF8 |
| hsa-miR-367 | RPL36A, RPS19 |
| hsa-miR-373 | TUBB2C, CLIC1 |
| hsa-miR-376b | GPI |
| hsa-miR-379 | DBI, NDUFV1, C19orf56, ARPC5L |
| hsa-miR-412 | COX6B1, HIST1H1C |
| hsa-miR-424 | AEBP1, VCAM1 |
| hsa-miR-425 | PPP1R14B, RPS17, ARPC5L |
| hsa-miR-429 | NME1, SNRPE |
| hsa-miR-448 | RPLP1, UQCRFS1 |
| hsa-miR-451 | LDHA, IFRD2 |
| hsa-miR-453 | PPP1R14B |
| hsa-miR-484 | CTSH |
| hsa-miR-485-5p | TUBB2C, COX7A2, COX7C, COX8A, GPI, NINJ1, TOR3A, FMNL1, IFRD2, TAGLN2 |
| hsa-miR-488 | CLIC1, SNRPE, UQCRFS1 |
| hsa-miR-492 | RPL35 |
| hsa-miR-505 | DUSP5, ARHGAP4, RPS10, SNRPD2, SNRPE, RPL14 |
| hsa-miR-510 | RPL35, DBI, RPS10, IFRD2 |
| hsa-miR-512-3p | GPI |
| hsa-miR-515-5p | CLIC1, NME1, RPL24, RPS24 |
| hsa-miR-520b | CLIC1, NME1 |
| hsa-miR-521 | HIST1H1C, CCND2 |
| hsa-miR-522 | ARPC5L |
| hsa-miR-523 | ARPC5L |
| hsa-miR-526b | IFRD2 |
| hsa-miR-549 | RPL35, SNRPE |
| hsa-miR-552 | HIGD2A, CCNB1IP1, RPS16, UCK2 |
| hsa-miR-553 | CLIC1, SNRPE, HIST1H2AC |
| hsa-miR-562 | DBI, CCNB1IP1 |
| hsa-miR-563 | COX7A2, COX7C, C15orf24, BCL3, RPS10, IFRD2 |
| hsa-miR-569 | NME1, RPS24 |
| hsa-miR-570 | TUBB2C |
| hsa-miR-578 | RPL35, GAPDH, C15orf24, RPLP1 |
| hsa-miR-585 | PPP1R14B, ARHGAP4, RPS16, SNRPB, UQCRFS1 |
| hsa-miR-596 | JUND, IFRD2 |
| hsa-miR-599 | ARID5A, DBI, NME1 |
| hsa-miR-600 | NME1, HIST1H2AC |
| hsa-miR-603 | DRAP1, GPI, LDHA, C15orf24, RPS21, ARPC5L |
| hsa-miR-606 | CLIC1, DBI, RPS12, RPS21 |
| hsa-miR-607 | HIGD2A, UQCRFS1 |
| hsa-miR-609 | ARID5A, GPI, C19orf56 |
| hsa-miR-613 | JUND |
| hsa-miR-614 | ARPC5L |
| hsa-miR-617 | RPL36, GAPDH |
| hsa-miR-622 | UQCRFS1, IFRD2 |
| hsa-miR-645 | CLIC1, C19orf56, PER1, CCNB1IP1, RPS12, RPS17, FMNL1, IFRD2 |
| hsa-miR-647 | PPP1R14B |
| hsa-miR-650 | C19orf56 |
| hsa-miR-652 | HIGD2A, PPP1R14B, RPS19 |
| hsa-miR-657 | DDT, EHD4, C19orf56, PER1, SH3GL1, TUFM |
| hsa-miR-658 | CLIC1, COX6B1, HIGD2A, HIST1H1C, NDUFV1 |
| hsa-miR-661 | ARID5A, CLIC1 |
| hsa-miR-662 | HIGD2A |
| hsa-miR-95 | COX6B1, GAPDH, C19orf56, SNRPD2, SNRPE, TXN, C4orf31 |
| hsa-miR-99b | HIST1H1C |
