# Supplementary material for: Measures of Association for Identifying MicroRNA-mRNA Pairs of Biological Interest
Source: PLoS One. 2012 Jan 11;7(1):e29612. doi: 10.1371/journal.pone.0029612 (PMC3256172; doi:10.1371/journal.pone.0029612)
Supplement: Appendix S2 — An illustration of the results obtained using UD association measure as the number of conditions varies. (DOC) [file pone.0029612.s006.doc]

If the miRNA-mRNA data correspond to a small number of conditions, then the unmatched data (UD) association measure is not suitable for identifying the significant miRNA-mRNA pairs. To demonstrate this, we assume that the discretized miRNA and mRNA data are available for 10 conditions, namely C_1_–C_10_ (Table 1). Here, −1 represents miRNA/mRNA underexpression with respect to the reference, 1 represents miRNA/mRNA overexpression with respect to the reference, and 0 represents no change in expression with respect to the reference.

**Table 1** Discretized miRNA and mRNA expression values

|  | **C_1_** | **C_2_** | **C_3_** | **C_4_** | **C_5_** | **C_6_** | **C_7_** | **C_8_** | **C_9_** | **C_10_** |
| --- | --- | --- | --- | --- | --- | --- | --- | --- | --- | --- |
| **miRNA** | −1 | −1 | 1 | 0 | 0 | −1 | −1 | −1 | −1 | 0 |
| **mRNA** | −1 | −1 | −1 | 0 | 0 | 1 | 1 | 1 | 1 | 0 |

Our null hypothesis is that there is no association between the miRNA-mRNA pair. We begin by populating a 3 × 3 contingency table (Table 2) using miRNA and mRNA data. Under the null hypothesis, the entries in this contingency table follow a multinomial distribution, which can be approximated by a χ^2^ distribution (degrees of freedom = 4) if the number of conditions is large. Next, we calculate the *p*-value for observing this table under the null hypothesis. Typically, we control for Type I error at a significance level of 0.05, which implies that we reject the null hypothesis when the *p*-value is less than 0.05. If the null hypothesis is rejected, then we consider the association between the miRNA-mRNA pair to be significant.

**Table 2** Generic contingency table

|  |  |  | **mRNA** |  |
| --- | --- | --- | --- | --- |
|  |  | −1 | 0 | 1 |
|  | −1 | a_11_ | a_12_ | a_13_ |
| **miRNA** | 0 | a_21_ | a_22_ | a_23_ |
|  | 1 | a_31_ | a_32_ | a_33_ |

The calculation of *p*-value requires the expected probabilities of a_11_–a_33_ under the null hypothesis. Since the miRNA and mRNA can take three values (i.e., −1, 0, and 1), in the simplest case, all the expected probabilities are equal to 1/9 and the *p*-values mentioned below correspond to the simplest case.

To illustrate the differences in results obtained using the UD association measure as the number of conditions varies, we considered two scenarios – (i) the miRNA and mRNA data correspond to all 10 conditions and (ii) the data correspond to two arbitrarily chosen conditions, C_8_ and C_9_. For Scenario (i), the *p*-value for the contingency table (Table 3), was 0.02. Since one typically rejects the null hypothesis if the *p*-value is less than 0.05, we concluded that the association between the miRNA-mRNA pair was significant. Therefore, an analysis of miRNA-mRNA data based on 10 conditions suggested that the pair was of potential biological interest. For Scenario (ii), the *p*-value for the contingency table (Table 4) was 0.11 (a value larger than 0.05), which suggested that the association between the miRNA and mRNA was not higher than that expected by chance. The large *p*-value could be reflecting the truth (i.e. there is no association between the miRNA and mRNA) or it could be the result of a lack of power.

**Table 3** Contingency table obtained using conditions C_1_ – C_10_

|  |  |  | **mRNA** |  |
| --- | --- | --- | --- | --- |
|  |  | −1 | 0 | 1 |
|  | −1 | 2 | 0 | 4 |
| **miRNA** | 0 | 0 | 3 | 0 |
|  | 1 | 1 | 0 | 0 |

**Table 4** Contingency table obtained using conditions C_8_ and C_9_

|  |  |  | **mRNA** |  |
| --- | --- | --- | --- | --- |
|  |  | −1 | 0 | 1 |
|  | −1 | 0 | 0 | 2 |
| **miRNA** | 0 | 0 | 0 | 0 |
|  | 1 | 0 | 0 | 0 |

To explore this further, we assumed the number of conditions to be two and considered all combinations of discretized miRNA and mRNA expression values. Since a miRNA/mRNA can take three distinct values, the total number of combinations was 81 (i.e. 3^4^). These 81 combinations corresponded to 45 distinct contingency tables and the smallest *p*-value was 0.11. Since all the *p*-values were larger than 0.05, even if there was a genuine association between the miRNA and mRNA, the UD measure would suggest otherwise. This implies that the UD association measure has low power when the number of conditions is two.
